# A Robust Model System for Retinal Hypoxia: Live Imaging of Calcium Dynamics and Gene Expression Studies in Primary Human Mixed Retinal Culture

**DOI:** 10.3389/fnins.2019.01445

**Published:** 2020-02-07

**Authors:** Shahna Shahulhameed, Sarpras Swain, Soumya Jana, Jay Chhablani, Mohammad Javed Ali, Rajeev R. Pappuru, Mudit Tyagi, Sushma Vishwakarma, Nanda Sailaja, Subhabrata Chakrabarti, Lopamudra Giri, Inderjeet Kaur

**Affiliations:** ^1^Brien Holden Eye Research Centre, LV Prasad Eye Institute, Hyderabad, India; ^2^Department of Chemical Engineering, Indian Institute of Technology, Hyderabad, India; ^3^Department of Electrical Engineering, Indian Institute of Technology, Hyderabad, India; ^4^Medical Retina and Vitreoretinal Surgery, University of Pittsburgh School of Medicine, Pittsburgh, PA, United States; ^5^Govindram Seksaria Institute of Dacryology, LV Prasad Eye Institute, Hyderabad, India; ^6^Smt. Kanuri Santhamma Center for Vitreo Retinal Diseases, LV Prasad Eye Institute, Hyderabad, India

**Keywords:** retina, glia, calcium spiking, neurons, hypoxia, neurodegeneration, inflammation

## Abstract

The detailed mechanisms underlying oxidative stress that leads to neuroinflammation and neurodegeneration in retinal vascular conditions, including diabetic retinopathy, retinopathy of prematurity etc., remain largely unexplored mainly due to a lack of suitable disease models that can simulate the inherent neuron–glia interactions in human retina. Specifically, establishment of a mixed retinal culture (MRC) containing both neuron and glial cell types remains a challenge due to different conditions required for their optimal growth and differentiation. Here, we establish a novel primary MRC model system containing neurons, astrocytes, Müller glia, and microglia from human donor retina that can be used to study the neuromodulatory effects of glial cells under the stress. The cell characterization based on immunostaining with individual cell type–specific markers and their presence in close vicinity to each other further underscores their utility for studying their cross talk. To the best of our knowledge, this is the first instance of an *in vitro* model obtained from human donor retina containing four major cell types. Next, we induce hypoxic stress to MRC to investigate if hypoxia activated neuroglia modulates altered gene expression for inflammatory, apoptotic, and angiogenic markers and Ca^2+^ transients by live cell imaging. Further, we performed *k*-means clustering of the Ca^2+^ responses to identify the modification of clustering pattern in stressed condition. Finally, we provide the evidence that the altered Ca^2+^ transient correlates to differential expression of genes shown to be involved in neuroinflammation, angiogenesis, and neurodegeneration under the hypoxic conditions as seen earlier in human cell lines and animal models of diabetic retinopathy. The major features of the hypoxic conditions in the proposed human MRC model included: increase in microglia activity, chemokine and cytokine expression, and percentage of cells having higher amplitude and frequency of Ca^2+^ transients. Thus, the proposed experimental system can potentially serve as an ideal *in vitro* model for studying the neuroinflammatory and neurodegenerative changes in the retina and identifying newer drug targets.

## Introduction

Neuroglia interactions in the retina are known to play a crucial role for maintaining retinal homeostasis. Abnormalities in glial cell activation disrupt the homeostasis, leading to inflammation, neovascularization, and compromised retinal functions, thereby causing neurodegenerative diseases such as retinopathy of prematurity (ROP), Age-related macular degeneration (AMD), glaucoma, and diabetic retinopathy. Cytosolic calcium (Ca^2+^) plays a key role in regulation of homeostasis in the retina, and its waves are known to maintain glia–astrocyte, astrocyte–astrocyte, as well as astrocyte–neuron communication. Generally, glial cells are present in close contact with the neurons, and the neuronal activity has been shown to induce rise in intracellular Ca^2+^ levels in glia (Newman, 2005). While Ca^2+^ signaling has been studied in primary cultures of rat retina, mouse tissue slices, and pig retina (Pereira Tde et al., 2010; Rosa et al., 2015; Agte et al., 2017), a systematic and quantitative analysis of it in the human retina with multiple cell types remains elusive. Specifically, there is a paucity of cell-based models to obtain the baseline functionality in the form of Ca^2+^ spiking patterns in primary human retina including both glial cells and neurons. Furthermore, the retina being a highly complex 3D structure with multiple cell types arranged in a well-defined pattern, it is rather challenging to establish an *in vitro* disease model for drug screening studies. Therefore, recent studies focus on optimization of culture conditions for culturing of two or more cell types in order to simulate complex *in vivo* situation (Skytt et al., 2016; Park et al., 2018).

Cytosolic calcium signaling in glial cells is known to be significantly altered for various eye diseases (Pereira Tde et al., 2010; Crish and Calkins, 2011). Specifically, in the case of neurodegeneration, the increase in basal Ca^2+^ level and augmented Ca^2+^ transients in astrocytes cause neurotoxicity (Kuchibhotla et al., 2009). It has also been indicated that the activation of microglia and associated increase in Ca^2+^ flux may kill the neurons, as observed in mouse retinal degenerations (Yu et al., 2015; Zhao et al., 2015). An increased level of oxidative stress and inflammation in retinal microenvironment often enhances retinal neurodegeneration under varied retinal pathology (Rohowetz et al., 2018). Hence, the effect of hypoxia has gained considerable interest as a mediator of retinal injury and inflammation (Arden and Sivaprasad, 2011). While the increase in Ca^2+^ under hyperglycemic conditions is known to cause neurodegenerative conditions (Shin et al., 2014), there are limited investigations on the study of hypoxia-mediated modulation of Ca^2+^ dynamics in retina.

In general, the existing studies on hypoxia in the retina are restricted in mice (Brahmachari et al., 2006; Rosa et al., 2015) and pigs (Hainsworth et al., 2002; Acharya et al., 2017), and none of these provides a suitable model for studying the human retina (Hartung, 2008). The studies on immortalized cell lines are usually derived from tumor cells that suffer from the loss of original tissue specificity and phenotypes with multiple passages (Matteucci et al., 2015). While there are independent investigations on primary cultures of Müller (Puro, 2002), astrocytes (Barber et al., 2000), and microglia (Ibrahim et al., 2011), there are limited investigations focusing on co-culturing them together. The rationale behind developing such a co-culture model containing multiple glial cell types stems from the fact that the microglia–Müller glia cross talk acts as a critical mechanism in the modulation of retinal response to injury in the mouse models (Arroba et al., 2014).

The major challenge in generating a model for studying retinal neurodegeneration includes the inherent heterogeneity in cellular activity and induction of neuroinflammation through activation of microglia and astrocytes. Hence, we aim to establish a primary mixed retinal culture model containing neurons, astrocytes, microglia, and Müller glia resembling the major cellular composition of human retina. Further, in order to identify their interactions and changes in pattern in calcium dynamics under hypoxic conditions, we proposed studying the collective responses through time-lapse calcium imaging. Thus, in order to obtain a statistical model for the heterogeneity present in MRC, we performed clustering of Ca^2+^ transients obtained from primary mixed retinal cell culture under normal and oxidative stress conditions. Similar approaches of clustering and classification of Ca^2+^ spiking were implemented to study the effect of G-protein coupled receptors (GPCR) targeting drugs on Ca^2+^ response for rat hippocampal neurons (Swain et al., 2018).

First, we show that the proposed *in vitro* system contains four major types of retinal cells in the culture. We further assessed if there is a change in gene expression profiles under hypoxia and found significantly differential expression of proinflammatory and angiogenic genes and cytokines. Consistent with the previous studies on rat/human retina, our proposed model demonstrated an increase in ionized calcium-binding adaptor molecule 1 (IBA1) and glial fibrillary acidic protein (GFAP) protein levels and increased expression for hypoxia inducible factor 1α (*HIF-1*α), C-X-C chemokine receptor type 4 (*CXCR4*), Interleukin 1β (*IL1-*β), and vascular endothelial growth factor (*VEGF*). Further, this work shows that the clustering of calcium dynamics is significantly modulated under hypoxia. It also reveals that hypoxia induces an increase in the percentage of hyperactive cells that correlate with the activation of microglial cells obtained from spatial mapping of IBA1 expression in the mixed population. Our model shows reproducibility in gene expression and clustering pattern of Ca^2+^ response across different cultures obtained from various human subjects. Thus, the co-culture model presented here can be regarded as a robust model for retinal hypoxia that can be used for studying the pathological mechanisms involved in various retinal vascular and neurodegenerative conditions.

## Materials and Methods

### Preparation of Human Mixed Retinal Cell Cultures

The study adhered to the tenets of the declaration of Helsinki and was approved by the institutional review board of LV Prasad Eye Institute, Hyderabad. Donor retinas from cadavers as well as from patients due to conditions such as staphyloma and open globe injury were used to establish a primary mixed retinal cell culture system. The cadaveric donor eyes were collected (within 24 hours of death) in sterile moist glass bottles from Ramayamma International Eye Bank, LV Prasad Eye Institute, and washed with sterile phosphate buffered saline (PBS) containing 2X concentrations of penicillin and streptomycin. The retinal tissues were removed using a pair of sterile forceps from the donor eye by making a posterior cut and washed gently with 1X PBS. The enucleated eyeballs from patients were collected after obtaining written informed consent and immediately transported to the lab on ice. The retina was removed from these eyeballs similar to the cadaveric eyes. The retinal tissues collected from either of the sources were washed gently with 1X PBS to remove the RPE and choroid pigments. The tissue was then chopped into small pieces using a sterile surgical blade. The chopped tissues were again washed with 1X PBS and treated with 1X trypsin EDTA (0.25%) for a period of 15–20 min at 37°C. Trypsin activity was arrested by adding complete DMEM (DMEM + 10% FBS + 1% penicillin–streptomycin) and centrifuged at 1,000 rpm for 3 min. The dissociated pieces were collected and resuspended in 2 ml PBS and gently triturated with a P1000 pipette tip to further obtain a suspension of cells. The suspensions of the cells were then passed through a 70-micron-size cell strainer to remove undigested pieces of tissues, if any. The cells were collected after the centrifugation and resuspended in DMEM containing 10% serum and 1% antibiotics. The cells were seeded in a sterile tissue culture grade T-75 mm flask and kept undisturbed for 7 days under standard cell culture conditions followed by changing medium every 3 days. 1ng/mL of granulocyte macrophage cytokine stimulating factor GM-CSF [REC. HUMAN GM-CSF 10 UG BIOSOURCE (TM), PHC2015], was added to the culture until the first medium change.

### Immunofluorescence for Retinal Cell Characterization

Immunofluorescence was done to characterize the cells in MRC. Briefly, the cells (approximately 5,000 cells/ml) were seeded on a sterile glass coverslip and allowed to attain 70–80% confluency. The cells were fixed with 4% formaldehyde in PBS for 10 min at room temperature. The cells were washed with 1X PBS and permeabilized with 0.5% Triton X-100 in PBS for 10 min. This was followed by incubation with blocking buffer consisting of 2% BSA in PBS for 1 h at room temperature. The primary antibodies were diluted with blocking buffer and added to the cells for overnight incubation at 4°C. The primary antibodies were used for identification of cells in the MRCs including mouse anti–ionized calcium-binding adaptor molecule 1 (for microglia, Abcam, Catalog No. ab178680), rabbit anti–glial fibrillary acidic protein (for astrocytes, Catalog No. Dako, Z0344), rabbit anti nestin (for neuronal progenitor cells, Millipore, Catalog No. ABD 69), rabbit beta-III tubulin (β-III tubulin; for neurons, Abcam, Catalog No. ab18207), and rabbit anti glutamine synthetase (GS; for Müller glia, Abcam, Catalog No. ab176562). The cells were washed thrice with 1X PBS followed by incubation for 45 min at room temperature with secondary antibodies (diluted in blocking buffer) Alexa Fluor 488 conjugated anti rabbit (Life Tech, Catalog No. A11008), Alexa Fluor 594 conjugated anti rabbit (Life Tech, Catalog No. A11012), and Alexa Fluor 594 conjugated anti mouse (Life Tech, Catalog No. A11005). The cells were washed thrice with 1X PBS, mounted with SlowFade Gold Antifade containing DAPI (Life Technologies, Ref. S36939), and scanned using an EVOS fluorescent microscope.

### Cell Viability Estimation and Hypoxia Induction

The cells from earlier passages (P1 and P2) were used for the experiment. The cell viability was estimated using an Alamar blue dye–based assay using different concentrations of cobalt chloride (CoCl_2_; Sigma Aldrich, Catalog No. C-8661-25G). Briefly, 2,000 cells were seeded on a 96-well plate and allowed to attain 70–80% confluency. Prior to the exposure of stress, the complete DMEM was replaced with serum free medium and incubated for 6 h. The cells were then treated with 100 and 150 μM CoCl_2_ for inducing hypoxia for a period of 24 h in serum free medium. Alamar blue reagent (10 μl) (Life Technologies, Catalog No. DAL1025) was added onto the cells containing 100 μl of medium and kept for incubation at standard cell culture conditions for 3 h. DMEM with Alamar blue served as blank. The absorbance of the medium was measured; the blank values were subtracted from cells’ absorbance value; and the percentage of viability and significance were calculated.

Once the optimization for hypoxia drug concentration was achieved, 15,000 cells were seeded on a glass coverslip and allowed to grow for 70–80% confluency. CoCl_2_ (150 μM) was used for the treatment for 24 h in serum-deprived medium. The cells deprived of serum but not exposed to stress for the same duration were used as control.

### Quantitative Gene Expression Analysis by Real-Time PCR

Gene expression by PCR was done for characterizing the cultured retinal cells as well as to measure the expression of genes under hypoxia. In brief, the total RNA was extracted from the retinal cells before and after the stress induction by TRIzol method. Total RNA was reverse transcribed into cDNA using a Verso cDNA synthesis Kit (ThermoFisher Scientific, Catalog No. AB1453B) according to the manufacturer’s protocol. The primer sequences used for conventional PCRs are given in the Supplementary Table S1. In order to quantify the average mRNA expression in the entire MRC population, we performed quantitative real-time (qRT) PCR on an Applied Biosystems 7900 HT system for a total reaction volume of 20 μl. Reaction mixture (10 μ) included iTaq^TM^ Universal SYBR^®^ Green Supermix (BIO-RAD, Catalog No. 172-5121), 200- nM of primer, and cDNA. The relative measure of the concentration of the target gene (Ct) was calculated by using software SDS 2.4. Analysis of gene expression changes was done using the 2^–ΔΔ*Ct*^ method. Statistical analyses were performed using 2^–ΔΔ*Ct*^ ± SEM in three technical and biological replicates. The housekeeping gene β-actin was used as a normalizing control. The primer sequences used for qRT PCRs are given in the Supplementary Table S2.

### Protein Imaging and Quantification

In order to assess the glial hyperreactivity under hypoxia, we plan to compare the protein expression in GFAP-positive and IBA1-positive cells in control and stressed conditions. In order to achieve this, we performed immunocytochemistry and quantitative protein imaging. Large-scale imaging was performed using lasers with excitation at 405, 488, and 594 nm for DAPI, Alexa 488, and Alexa 594 with a Leica SP8 laser scanning confocal microscope with a 40X dry objective. In order to quantify the protein level in a large section of MRC, we acquired a panorama using the mosaicking technique for a field of view (1.8 × 1.8 mm) containing 10 × 10 square sections (each section of dimension 180 × 180 μm) with approximately 20% overlap. A hundred sections were stitched to obtain the spatial protein profiling for a large section by Leica LAS X Software. In order to show the representative images of GFAP and IBA1 expression under no stress and hypoxia, 3D imaging was performed through acquiring Z-stack images along the *z*-axis (total Z height = ∼12 μm, Z-stack thickness between each slice = 0.5 μm) with a 63× oil immersed objective. The fluorescence intensity corresponding to various regions of interest was acquired using Leica LAS X software. In order to quantify the glial reactivity in MRC, we further created a 3D surface plot of GFAP and IBA1 expression from the panorama images using ImageJ software.

### Time-Lapse Ca^2+^ Imaging of the Cells

In order to perform cytosolic calcium imaging, cells were loaded with 2 μM Fluo-4 (Molecular Probes, Life Technologies, Grand Island, NY, United States) for 30 min in Hank’s Balanced Salt Solution (HBSS) (Invitrogen, Life Technologies, Grand Island, NY, United States). The cells were then washed thrice with HBSS followed by fluorescence imaging (excitation: 488 nm) at 37°C. Time-lapse movies were acquired every 10 s, for 10 min, and raw data were analyzed with MATLAB (The MathWorks, Natick, MA, United States). An image segmentation algorithm, based on principal component analysis, was optimized for automated segmentation of cells present in MRC. The maximum amplitude of Fluo-4 intensity and Ca^2+^ spike count were computed for control and hypoxia and were represented through box plots. In order to perform the correction for the photobleaching effect, we used second-order polynomial fitting and estimation of coefficients.

### Data Analysis

The time course of Ca^2+^ transients obtained for 600 s was analyzed using MATLAB. The analysis was performed for all the segmented cells obtained from the live imaging video for respective conditions. In order to quantify the activity level in MRC, we obtained the raster plot via peak identification from the time course of Fluo-4 intensity. Although the cell size has not been accounted for to make any size-based correction, the Fluo-4 intensity was normalized with respect to basal-level Fluo-4 intensity for each cell (Swain et al., 2018). We performed *k*-means clustering based on Ca^2+^ spike count and Ca^2+^_*max*_ (maximum calcium amplitude) (number of clusters *k* = 4 for control condition). The automation in classification of cells yielded different types of cells, black and cyan cells with lower activity, green cells with moderate activity and red cells with highest activity. Here, we report the cells with higher amplitude and spike count as the hyperactive cells and the cells with moderate amplitude and spike count as moderately active cells. Furthermore, we used the boundaries obtained from the control condition as the reference to classify the calcium transients under the hypoxic condition. The relative percentages of four subpopulations were represented using stack bar plots. The data set corresponding to control and hypoxia was tested for normality using the Jarque–Bera test. As the majority of the data set were not normally distributed, we have used the Kruskal–Wallis test to study the effects of hypoxia on a mixed retinal population. Statistical tests were performed at the significance level of 0.05. In box plots, the results were presented in terms of median, interquartile range, and whiskers 10–90%. We also performed the Kruskal–Wallis test to check whether the percentages of cells corresponding to different clusters (having high, moderate, low, and no activity) are significantly different in the stressed condition compared to the no-stress condition.

Data sampling: In order to select data from multiple videos in an unbiased manner, 60% of the cells were randomly chosen from the MRC population (population size = 160, sampling repeated five times, size of each random sample = 90 cells) for clustering (Swain et al., 2018). All bar graphs were plotted to present mean ± SEM. The schematic representation of data analysis is given in Supplementary Figure S9.

## Results

### Culturing of Primary Mixed Retinal Cells and Characterization

**FIGURE 1:**
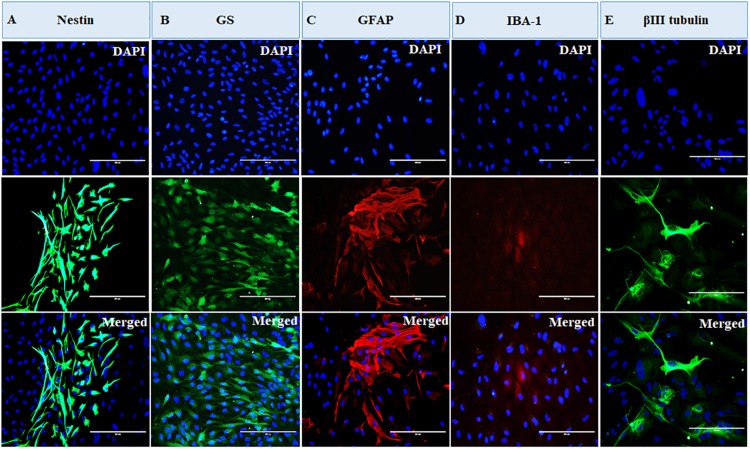
Immunofluorescence-based characterization of human primary mixed retinal cells. The representative images clearly show the presence of neurons and all types of glial cells: **(A)** cells expressing neuronal progenitor marker, nestin; **(B)** cells expressing Müller glia marker, glutamine synthetase (GS); **(C)** cells expressing astrocyte marker, glial fibrillary acidic protein (GFAP); **(D)** cells expressing microglial marker, ionized calcium-binding adaptor molecule 1 (IBA1); and **(E)** cells expressing neuronal marker, β-III tubulin (magnification 20×, scale bar 200 μm).

**FIGURE 2:**
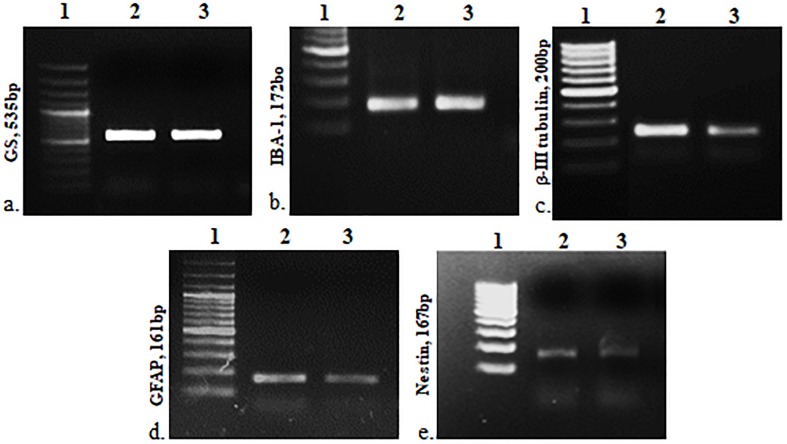
PCR-based characterization for cell type–specific markers: *GS, IBA1*, β*-III tubulin, GFAP* and *nestin*, respectively. 1, DNA ladder; 2, positive control (retina), and 3, mixed retinal cells (MRCs).

The cells were heterogeneous in nature, which was evident from their morphology. The dissociated retinal cell cultures started to adhere after 3–4 days and became confluent within 3–4 weeks in culture. Most importantly, the cells were having both neuronal and glial morphology with a network of processes (Supplementary Figures S1, S2). Immunofluorescent staining and PCR characterization were done for glial as well as neural populations of the cultured cells. Immunofluorescence of the cells in MRC clearly showed positive staining for neuronal progenitor marker nestin (Figure 1A), Müller glial marker GS (Figure 1B), GFAP for astrocytes (Figure 1C), microglia marker IBA1 (Figure 1D), and β-III tubulin (Figure 1E) for the neuronal population. Likewise, PCR-based characterization of the cells also confirmed the expression of genes specific to glial cells, neural progenitor cells, and mature neurons in the dissociated retinal culture (Figure 2).

### Reproducibility of Cell Population in MRC

**FIGURE 3:**
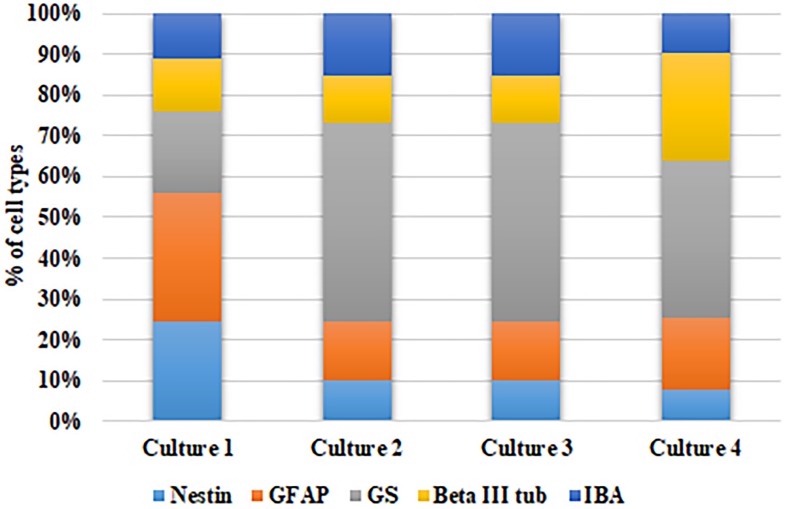
Analysis of model robustness in primary mixed retinal cultures obtained from different retinal tissues. The subpopulation percentages of four different cell types in MRC corresponding to samples from four retinal sources were calculated, and the percentage of each cell type is represented in the bar graph.

Further, to ascertain the robustness of this culture system and reproducibility of the cell types obtained, we have calculated the percentage of each cell type with respect to the total number of DAPI stained cells in each culture obtained from different cadaver retina samples. Figure 3 shows the stack bar representation of subpopulation percentages for each cell type in MRC corresponding to samples from four cadaver donor retinas. The result shows that the percentages of each cell type for the samples derived from different eyes are not significantly different (*p* > 0.05) (Figure 3).

### Cell Viability Under Hypoxic Stress

**FIGURE 4:**
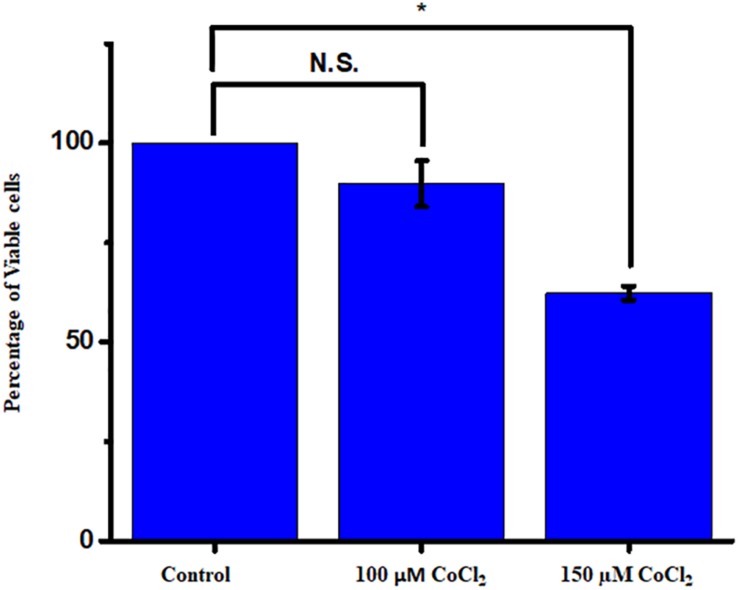
The mixed retinal cultures were treated with increasing concentration of CoCl_2_ for a period of 24 h. The viability was measured using Alamar blue–based dose-dependent cell viability assay (*N* = 3 biological and technical replicates). The data are represented as mean ± SEM (*n* = 3). N.S., not significant. *p < 0.05.

The cells were actively dividing until passage four, and the earlier passage of the cells (P1 and P2) was used for the experiment. Prior to the experiment, a PCR-based characterization was done for the cell-specific genes to ensure all major glial cell types and mature neurons in the culture (Supplementary Figure S3). The serum-deprived cells were exposed to different concentrations of CoCl_2_ for a time period of 24 h, and cell viability was measured by the Alamar blue method. We have used a concentration range from 100 to 250 μM of CoCl_2_. The cell viability of controls was always maintained as 100%. We have found a concentration-dependent reduction of cell viability under hypoxic treatment. The results showed a significant reduction in cell viability when the cells were treated with 150 μM (62.33 ± 1.71, *p* < 0.05) (Figure 4).

### Imaging of Ca^2+^ Spiking in a Mixed Retina Population

**FIGURE 5:**
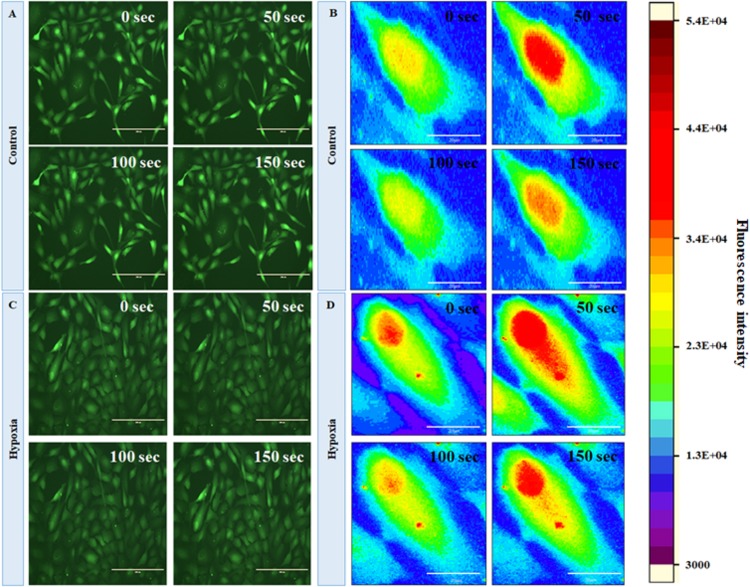
Fluorescent imaging of time course of cytosolic Ca^2+^C in human primary MRCs; representative time-lapse images for **(A)** control (no stress) and **(C)** hypoxia (150 μM CoCl_2_) (magnification 20×, scale bar 200 μm). Representative spatial intensity mapping of Ca^2+^ flux in single cell present in MRC: **(B)** control and **(D)** hypoxia. The results clearly identified that intracellular Ca^2+^ oscillates at variable frequencies for different cells in the MRC population (scale bar 20 μm).

**FIGURE 6:**
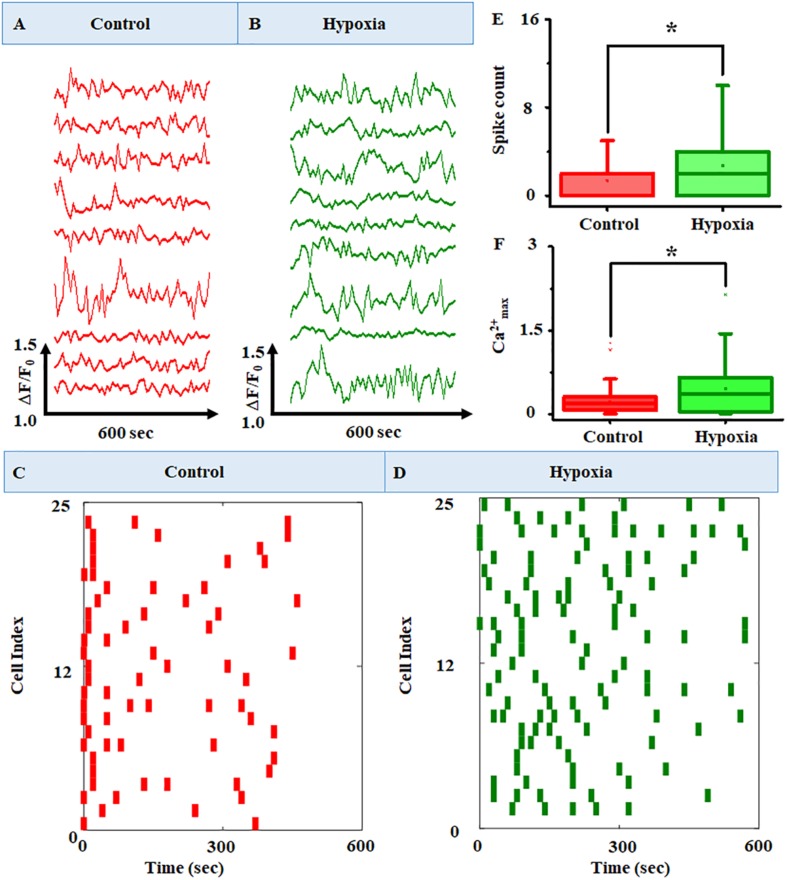
Representative raw plots of time course of cytosolic Ca^2+^ under **(A)** no stress and **(B)** hypoxia. The *x*-axis represents the change in fluorescence (ΔF/F0), and the *y*-axis represents the time course of the experiments. The results clearly identified intracellular Ca^2+^ oscillation at variable frequencies for different cells in the MRC population. A raster plot representing the network activity in MRC: **(C)** control and **(D)** hypoxia (*n* = 160). The raster plot showed that there are cells with a higher number of Ca^2+^ spike count in the case of hypoxia compared to the no-stress condition. Comparison of **(E)** Ca^2+^ spike count and **(F)** Ca^2+^_*max*_ between no-stress condition and hypoxia. Data were presented using a box plot (*p < 0.05; Kruskal–Wallis test).

In order to evaluate the changes in intracellular calcium level under hypoxia, we first characterized the basal-level cytosolic Ca^2+^ spiking in MRC. Figures 5A,B show the time-lapse imaging of cytosolic Ca^2+^ in MRC under the no-stress condition. (A movie file shows these details, Supplementary Movie S1). Next, we performed the time-lapse imaging of intracellular Ca^2+^ for hypoxia (Figures 5C,D; an additional movie file shows these details, Supplementary Movie S2). The spatial mapping of single-cell Fluo-4 intensity in the MRC population showed the Ca^2+^ spiking in a single cell under various conditions. The heat map representation of time-lapse Ca^2+^ responses provided prominent visualization of Ca^2+^ spiking (Figures 5B,D, and Supplementary Figure S4). The time-lapse videos were further processed by an image segmentation algorithm to acquire data, and the schematic diagram of the data acquisition process is shown in Supplementary Figure S5. The Ca^2+^ spiking pattern under no stress and hypoxia (Figures 6A,B) indicated that the intracellular Ca^2+^ oscillates at variable frequencies for different cells in the MRC population. Note that each of the cells in the whole population did not show Ca^2+^ spiking, indicating that there were some cells having relatively less activity.

In order to observe the Ca^2+^ spiking pattern in a large MRC population, we have plotted the raster plot for 160 cells (Figures 6C,D). The raster plot showed that there is an increase in Ca^2+^ spike count in the case of hypoxia compared to the no-stress condition. This was further validated using box plot representation (Figures 6E,F) showing the comparison of Ca^2+^ spike count and Ca^2+^_*max*_ between the no-stress condition and hypoxia. The box plot representation clearly indicated that hypoxia induces a significant increase in Ca^2+^ spike count in the MRC population (*p* < 0.05).

### Classification of Hypoxia-Mediated Modulation of Ca^2+^ Spiking

**FIGURE 7:**
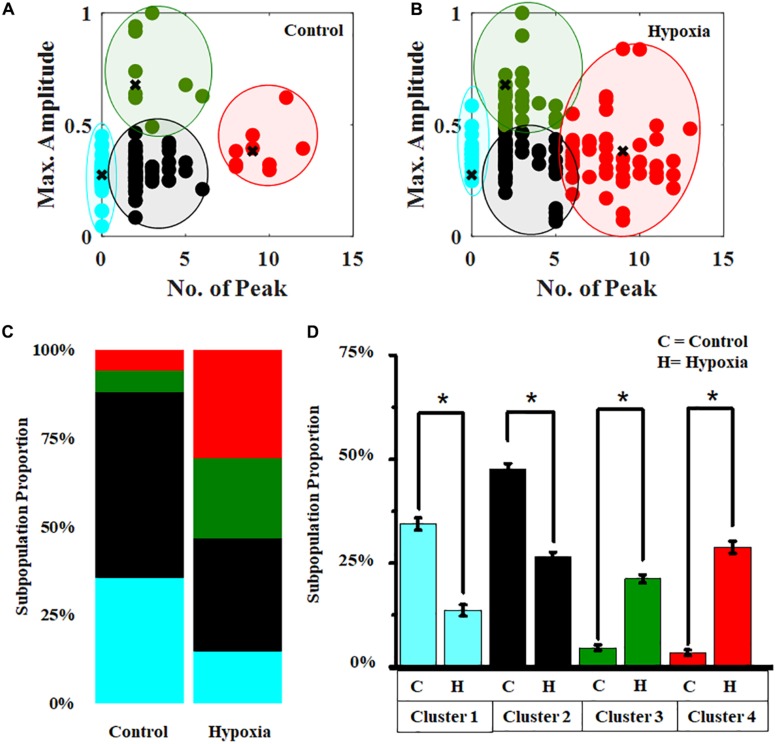
Hypoxia modulates the clustering pattern of Ca^2+^ spiking in MRC. Clustering pattern under **(A)** no stress (number of clusters, *k* = 4). **(B)** Classification of Ca^2+^ spiking under hypoxia. **(C)** Stack bars representing the subpopulation profiling of Ca^2+^ spiking corresponding to no stress and hypoxia. **(D)** Comparison of relative percentages of four clusters corresponding to no-stress and hypoxia conditions. The clustering was performed based on two features, Ca^2+^ spike count and Ca^2+^_*max*_. Red, hyperactive cells; green, cells with moderate activity; black and cyan, cells with lower activity. *p < 0.05; Kruskal–Wallis test. N.S., not significant.

In order to obtain a subpopulation profiling of the calcium spiking pattern present in the MRC population, we implemented the *k*-means clustering (Supplementary Figure S6) under the no-stress condition (Figure 7A). Since Ca^2+^ spike count and Ca^2+^_max_ can be used to characterize the neuronal activity, we chose these two features to perform the clustering of calcium spiking over time. The result showed that the cells can be grouped into various categories, hyperactive cells (high spiking, high amplitude >6 spikes in 10 min), cells with moderate activity (moderate amplitude, moderate spiking, 1–6 spikes per 10 min, Ca^2+^_max_ > 0.5), and cells with lower activity (low amplitude, moderate spiking, 0–6 spikes per 10 min, Ca^2+^_max_ < 0.5). Using these boundary constraints corresponding to two features for each subpopulation of MRC under the no-stress condition, we performed the classification of the Ca^2+^ spiking under hypoxia (Figure 7B). Further, we plotted the stack bars representing the relative percentages of each category (Figure 7C). The percentage of hyperactive cells and cells with moderate activity were found to be higher in hypoxia compared to the no-stress condition (*p* < 0.05) (Figure 7D). Moreover, the percentage of low active cells was significantly lower in the case of hypoxia compared to control (*p* < 0.05).

**FIGURE 8:**
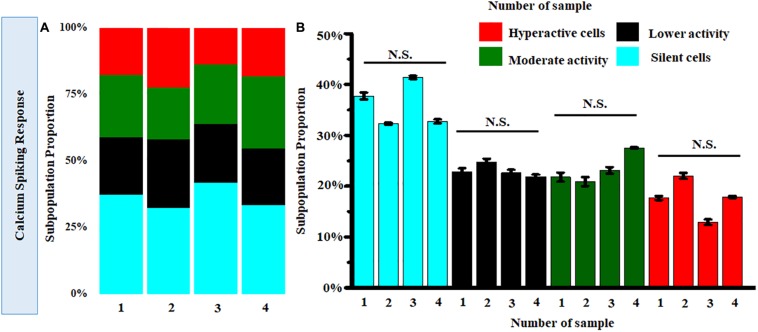
**(A)** Stack bar representation of subpopulation profiles of Ca^2+^ spiking corresponding to samples from four donor retinas. **(B)** Comparison of average relative percentages of various clusters across samples from four donor retinas. [Number of cells taken from each sample corresponding to single donor tissue = 160 (*p* > 0.05; Kruskal-Wallis test). N.S., not significant.]

Further clustering of Ca^2+^ spiking of MRC under normal conditions obtained from four donor retinas was performed. Figure 8A shows the stack bars representing subpopulation profiles of Ca^2+^ spiking corresponding to cells from each donor retina. This analysis showed that the percentages of each subtype are not significantly different across patients (*p* > 0.05) (Figure 8B).

### Quantitative Gene Expression Analysis by Real-Time PCR

**FIGURE 9:**
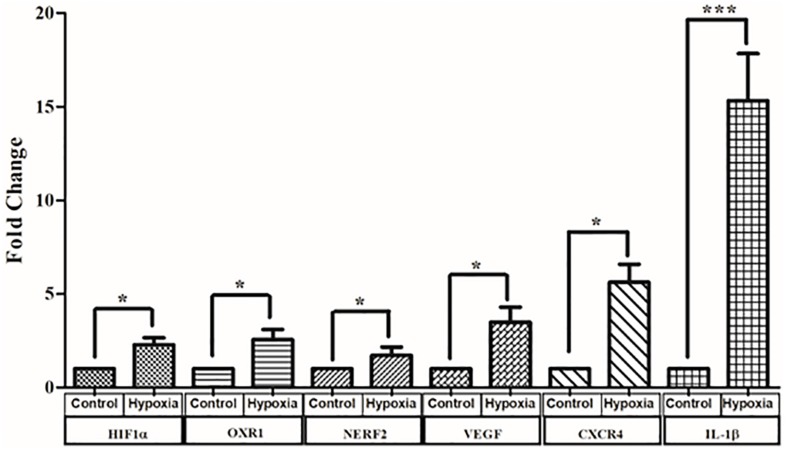
Real-time quantitative PCR analysis for genes involved in oxidative stress angiogenesis and inflammation and angiogenesis under hypoxia condition. The data are represented as mean ± SEM (*N* = 3). N.S., not significant. *p < 0.05, ***p < 0.001; Kruskal–Wallis test.

Cells exposed to 150 μM CoCl_2_ in serum-deprived medium to induce hypoxia and control cells were harvested after 24 h, and RNA was extracted. Real-time PCR was performed for three sets of heterogeneous cell cultures derived from three different retina sources. The expression of representative genes from different pathways known to be involved in DR pathogenesis including hypoxia signaling (*HIF1-*α, *NERF2*, and *OXR1*) inflammation (*IL-1*β, *IL-8*, and *C3*), angiogenesis (*CXCR4* and *VEGF*), and apoptosis (*BAX* and *Caspase 3*) was measured. (Out of 11 genes measured, the expression of six genes was found to be significantly upregulated in hypoxia; Figure 9). These include genes such as *HIF1-*α, which was found to be significantly upregulated by 2.28 ± 0.37-fold under hypoxia (*p* < 0.05). Likewise, the genes involved in oxidative stress response such as *OXR1* and *NERF2* were upregulated under hypoxia (*OXR1*: 2.56 ± 0.53, *p* < 0.05; *NERF2*: 1.7 ± 0.4, *p* < 0.05). Further, the angiogenic genes such as *VEGF* and *CXCR4* were upregulated 3.48 ± 0.8-fold, *p* < 0.05, and 6.89 ± 1.02-fold, *p* < 0.05, respectively. The expression of *IL-1*β was found to be 15.3 ± 2.5, *p* < 0.05, in hypoxia treated cells, even though the expression of other inflammatory genes such as *C3, IL-8* was found to be higher in hypoxic treatment, while this increase was not found to be significant (*C3*: 1.53 ± 0.05, *p* > 0.05; *IL-8*: 1.7 ± 0.4, *p* > 0.05). Likewise, the apoptotic markers *Caspase 3* and *BAX* showed an increased expression under hypoxia (*Caspase 3*: 2.26 ± 0.63, *p* > 0.05; *BAX*: 1.41 ± 0.3, *p* > 0.05).

### Quantitative Analysis of IBA1 and GFAP at Protein Level Under Hypoxic Conditions in Primary Mixed Retina Culture

**FIGURE 10:**
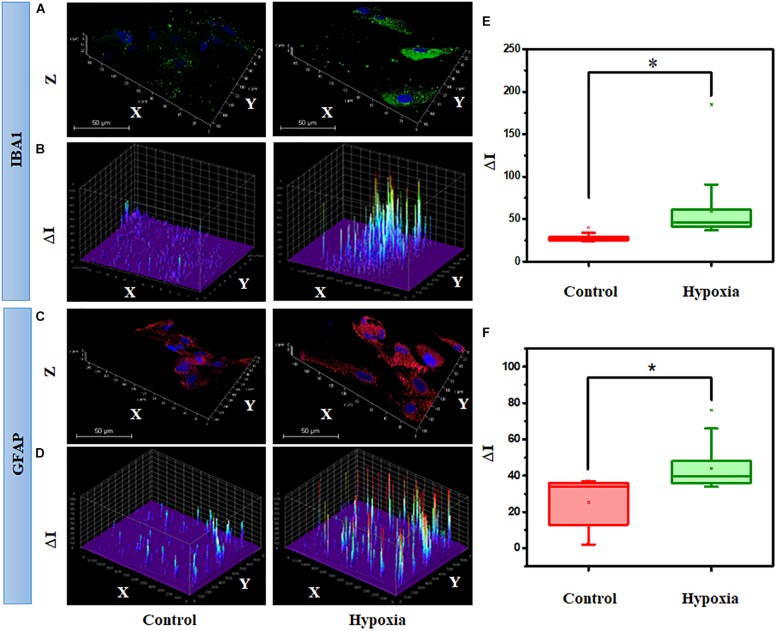
Hypoxia alter the spatial profiling of IBA1 expression in MRC. **(A)** Representative 3D images and **(B)** surface plot showing IBA1 expression under no stress and hypoxia (ΔI indicating the fluorescent intensity corresponding to protein level). **(C)** Representative 3D images and **(D)** surface plot showing the spatial profiling of GFAP expression under no stress and hypoxia. Quantitative analysis of protein expression was done in a large number of cells under each condition, represented in a box plot. **(E)** Comparison of IBA1 and **(F)** GFAP expression between no stress and hypoxia. ^∗^*p* < 0.05. N.S., not significant; Kruskal–Wallis test.

Next, we hypothesized that the expression of cell type–specific protein is increased in the MRC population when subjected to hypoxia. Since significant spatial heterogeneity was observed for various proteins in MRC, the protein expression was quantified through large-scale imaging using a confocal microscope. To examine the hypoxic injury on microglia and astrocytes, we analyzed IBA1 and GFAP expression from the panorama images. Figures 10A,C show the representative 3D images of IBA1- and GFAP-positive cells chosen from MRC under hypoxic injury. In order to assess the glial reactivity, we constructed a 3D surface plot corresponding to spatial profiling of IBA1 and GFAP under control and stress conditions (Figures 10B,D).

The spatial pattern shows the differential expression of IBA1 and GFAP under stress compared to the control condition, indicating the activation of microglia and gliosis, respectively, under injury. Next, we performed a quantitative analysis of protein expression in a large number of cells under each condition through box plots (Figures 10E,F). The result suggests that hypoxia induces a significant increase in IBA1 expression (2-fold) and GFAP (1.7-fold) (*p* < 0.05). In addition to this, we also evaluated the protein expression of GS under the hypoxic condition, however, there was no significant difference (*p* > 0.05, Kruskal–Wallis test) between the control and hypoxic conditions (Supplementary Figure S7). A double staining of GFAP and GS was also performed in retinal cells under control and hypoxic conditions. This identified a clear categorization of GFAP- and GS-positive cells in the culture (Supplementary Figure S8), and upon treatment, the GFAP level was found to be elevated in the cells, and there was no change in the expression of GS.

## Discussion

Glial cells are the supporting cells of the neural retina (Rubsam et al., 2018), and the homeostatic changes in the retina due to hypoxia or diabetes as seen in retinal vascular conditions like DR, ROP, etc., affect these supporting cells, which eventually leads to neurotoxic consequences such as glutamate excitotoxicity caused by Müller glia dysfunction (Ishikawa, 2013), aberrant activation of microglia, astrogliosis, etc. (Fischer et al., 2011). But there is a very little information available regarding neuroglia interaction in the retina and their interaction during the progression of retinal vascular and neurodegenerative diseases. Ca^2+^ signaling being the major intrinsic signaling system in the glial cells plays a vital role in angiogenesis, inflammation, and most importantly neuroprotection in the retina (Vecino et al., 2016). Hence, it is imperative to understand the changes in this intrinsic signaling system and their effect on neuronal damage under the stressed condition in a system that closely mimics the human retina.

The current study explores the synergistic activity of neurons, Müller glia, astrocytes, and microglia in the MRC under normal and stress conditions through clustering of calcium dynamics obtained from population-level calcium imaging, gene expression profiles, as well as quantitative protein expression studies. The major finding of the present study is that the induction of hypoxia significantly modulates the Ca^2+^ pattern in MRC along with an increase in IBA1 and GFAP levels in microglia and in macroglial cells, respectively.

Currently, with the advent of newer cellular and regeneration technologies, organoid cultures derived from iPSCs are more in focus for studying and modeling the complex retinal diseases (Hallam et al., 2018). These organoid cultures can mimic the *in situ* response and thereby provide a suitable platform for studying the complex cellular interactions and early developmental changes, however, during the process of their development, they undergo extensive genetic manipulations. Additionally, it requires high maintenance cost and longer duration for developing organoids reproducibly (Ho et al., 2018). Most importantly, organoids derived from iPSCs lack differentiation into essential retinal cell phenotypes including endothelial and microglial cells (Achberger et al., 2019). Since microglial cells are known to modulate the response to oxidative stress and injury, using iPSCs derived cellular model may not be appropriate for hypoxia studies. The proposed primary mixed culture system developed in this study, therefore, provides an advantage over organoid-based models, primarily owing to no genetic manipulation, being easy to work, cost effectiveness, and most importantly, having the major retinal cell types being represented uniformly and reproducibly across all the cultures. However, obtaining sufficient human retina tissue without any degenerative changes and within 24 h in sterile conditions for culturing could be challenging.

In order to establish the *in vitro* model for studying neuron–glia interactions under hypoxic conditions, it is essential to study both neuron and glial cell types in close proximity so that they can interact with each other. Since the response to any stress/injury is a function of different cell types present, we optimized the culture conditions such that it enables simultaneous growth of four cell types. Also, no trophic and other growth factors were added to selectively differentiate them into specific cell types. While it would have been worthwhile to have endothelial cells too in the same culture, the required conditions for the same made it difficult to have them cultured along with neuron and glial cell types. Due to a lack of enough cells for a flow cytometry–based counting, the percentage of each cell type in different MRC cultures were calculated (Figure 3) and found to be similar across cultures, implying the robustness of this model. However, these cell type–specific ratios may change post-hypoxia induction based on the response to hypoxia by each cell type. Further, we preferred to use cells only from early passages to maintain them close to the original phenotype. The gene expression analysis and functionality imaging together show that the proposed MRC obtained from the human eye is robust and reproducible and has the potential to be used for drug screening.

HIF-1α is the key regulator mediating the responses to hypoxia. Under normal oxygen tension conditions, HIF-1α protein turnover is very quick due to the action of prolyl hydroxylases, that promote its binding to the Von Hippel–Lindau protein, ubiquitination, and subsequent proteosomal degradation. Exposure with CoCl_2_ blocks the catalytic activity of prolyl hydroxylases, leading to the stabilization and accumulation of the HIF-1α protein, thereby creating an intracellular hypoxia-like state (Cervellati et al., 2014). Stabilization of HIF-1α typically promotes the synthesis of reactive oxygen species (ROS), which further, based on their intracellular concentration, modulate the transcription of genes involved in cell proliferation, differentiation, and death (Shu et al., 2019). The major findings of the present study suggest that hypoxia plays a significant role in regulation of inflammation, cellular apoptosis, and vascular changes as seen in ischemic complications of the retina such as DR.

Since average protein expression across multiple cell types present in the mixed culture may not capture the changes present in specific cell types, we performed quantitative imaging for microglia and macroglia using their specific marker proteins IBA1 and GFAP respectively, under control and stressed conditions. The quantitative comparison of cytokine, apoptotic, and inflammatory gene expression in MRC upon the induction of hypoxic conditions also showed an increased expression of the major known genes associated with DR pathogenesis (Kowluru and Odenbach, 2004; Yan and Su, 2014).

In order to identify the fraction of hyperactive and silent cells, *k*-means clustering of calcium spiking was performed (Swain et al., 2018). The clustering study shows that hypoxia induced an increase in the percentage of hyperactive cells. Since the Ca^2+^ spiking patterns obtained from the mixed culture were found to be highly heterogeneous, the basal-level response in the control condition was categorized into various types. However, it would be strategic to determine the individual Ca^2+^ spiking signature specific to neuron, astrocyte, Müller, and microglia cells in the MRC. This would require measuring of the calcium spiking in live cells using the Fluo-4 dye and staining the respective cell types with specific protein markers. Calcium imaging along with live markers for each cell type such as neurons and astrocytes may yield better information on cell-specific responses in mixed retinal cells, though homogenous transfection of primary cells remains a challenge (Peri and Nüsslein-Volhard, 2008; Guo et al., 2017). In our model, we observed a significant modification in functionality through classification of calcium spiking under stressed conditions. However, future studies may include the investigation on the calcium channels and GPCRs involved in the process using channel inhibitor and GPCR targeting drugs. Further, measurement of glutamate and ROS may provide us insight into whether an excessive stimulation of glutamate receptors results in an uncontrolled intracellular Ca^2+^ flow in neurons as a consequence of oxidative stress.

## Conclusion

We report that the proposed model based on a human MRC system provides a significant improvement over current *in vitro* models based on individual cells. Our *in vitro* model reproducibly showed underlying key pathological changes as seen in the retina (Ca^2+^ activation and major signaling/pathways) under hypoxia mimicking diabetic retinopathy and other retinal vascular diseases. Further optimization of the culture conditions so as to include endothelial cells in this primary mixed retinal cell culture model is underway and would allow investigation of the neuromodulatory effects of glial cells on the angiogenesis in the retina. Although a 3D culture model may yield a better understanding of neurodegeneration (Park et al., 2018), a 2D study on a mixed system subjected to stress conditions can be used for drug testing studies. Moreover, measurement of calcium spiking and classification can be used for estimation of the neuronal activity and underlying inflammation in the retina.

## Data Availability Statement

All data generated or analyzed during this study are included in this published article and its Supplementary Material.

## Ethics Statement

The studies involving human participants were reviewed and approved by institutional review board, LV Prasad Eye Institute, Hyderabad, India Ref. no. LEC 02-14-029. The patients/participants provided their written informed consent to participate in this study.

## Author Contributions

IK and LG conceived the idea, wrote the protocol and served as principal investigators. SC, JC, MT, RP, MA, SJ, and SC were co-investigators. SSh performed most of the cell culture work and data analysis. SV performed cell culture work. SSw and NS performed analysis for the Ca^2+^ imaging data. SSh, SSw, IK, and LG analyzed the data and wrote the manuscript. All authors revised the manuscript and approved the submitted version.

## Conflict of Interest

The authors declare that the research was conducted in the absence of any commercial or financial relationships that could be construed as a potential conflict of interest.

## Supplementary Material

FIGURE S1 Representative phase contrast images of cells cultured from retina of human cadaveric/enucleated eyes. **(A)** Morphology of the cells after 4th, 8th, and 16th days of culture at P0 stage. The cells showing the characteristic morphology of glial and neuronal type cells at day 16 confirm the heterogeneous retinal cell types in culture. **(B)** Morphology of cells in the MRC at first, second, and third passages confirming the culture system is able to maintain these heterogeneous populations until third passage (Magnification 10×, Scale bar 200 μm).

FIGURE S2 Representative immunofluorescent images of cells in Mixed retinal culture. The images clearly showing interaction of different cell types in the developed culture system. Panel showing the co-staining of **(a)** vimentin (Müller glia) and GFAP (Astrocytes), **(b)** IBA-1 (Microglia) and GS (Müller glia), **(c)** β-III tubulin (Neurons) and GFAP (Astrocytes), **(d)** GS (Müller glia) and GFAP (Astrocytes) (Magnification 20×, scale bar, 200 μm).

FIGURE S3 Gene expression of neuron and glial cell specific markers from three different retinal donors’ tissue at P1 and P2 passages, (A, B, and C represents cells cultured from three different retinal tissues and 1 and 2 represents P1 and P2 passages).

FIGURE S4 Time lapse images of the spatial intensity mappings of cytosolic calcium transients in human primary mixed retinal culture **(A)** no stress **(B)** hypoxia (Magnification 20×, Scale bar 200 μm).

FIGURE S5 Workflow representing various steps consisting of data acquisition, automated cell segmentation, cell labeling and data processing from the raw time-lapse videos.

FIGURE S6 k-means clustering of Ca^2+^ spiking in control MRC **(A)** Raster plots representing the network activity in MRC **(B)** Clustering of Ca^2+^ spiking train in a MRC population using two features, Ca^2+^ spike-count and maximum Ca^2+^ spiking amplitude (Ca^2+^_max_) **(C)** Raster plot showing the clustering pattern in MRC population **(D)** Identification of optimal number of clusters for the Ca^2+^ spiking train using Davies-Bouldin index.

FIGURE S7 **(A)** GS expression in MRC under no stress and hypoxia **(B)** Surface plot showing GS expression under no stress and hypoxia **(C)** Comparison of GS expression between no stress and hypoxia. N.S.: not significant.

FIGURE S8 Representative immunofluorescent images of GS and GFAP in cells under **(a)** control and **(b)** hypoxic conditions. (Magnification, 20×, Scale bar- 200 μm).

FIGURE S9 A flow chart describing the detailed summary of the Ca^2+^ imaging data analysis.

TABLE S1 Nucleotide sequences of primers used in conventional PCR.

TABLE S2 Nucleotide sequences of primers used in quantitative Real time PCR.

VIDEOS S1, S2 Measurement of intracellular Ca^2+^ transient in MRC using EVOS microscope (magnification 20X). Movie files show the Ca^2+^ spiking corresponding to no stress level **(Movie S1)** and Hypoxia **(Movie S2)** Spiking response was measured for 600 s.
