# Supplementary material for: A Robust Model System for Retinal Hypoxia: Live Imaging of Calcium Dynamics and Gene Expression Studies in Primary Human Mixed Retinal Culture
Source: Front Neurosci. 2020 Feb 7;13:1445. doi: 10.3389/fnins.2019.01445 (PMC7020445; doi:10.3389/fnins.2019.01445)
Supplement: FIGURE S1 — Representative phase contrast images of cells cultured from retina of human cadaveric/enucleated eyes. (A) Morphology of the cells after 4th, 8th, and 16th days of culture at P0 stage. The cells showing the characteristic morphology of glial and neuronal type cells at day 16 confirm the heterogeneous retinal cell types in culture. (B) Morphology of cells in the MRC at first, second, and third passages confirming the culture system is able to maintain these heterogeneous populations until third passage (Magnification 10×, Scale bar 200 μm). [file Data_Sheet_1.PDF]

Supplementary File

Table S1. Nucleotide sequences of primers used in conventional PCR

Gene	Forward	Reverse	Annealing Temp.	References
GS	ATGCTGGAGTCA AGATTGCG	TCATTGAGAAGACAC GTGCG	60˚C	(Lawrence et al., 2007)
IBA-1	GACCTTAATGGA AATGGCGATA	ATCTCTTGCCCAGCA TCATC	58˚C	(Orsmark et al., 2007)
Nestin	GAAACAGCCATA GAGGGCAAA	TGGTTTTCCAGAGTC TTCAGTGA	60˚C	Primer Bank ID 35019a2
GFAP	CCTCTCCCTGGCT CGAATG	GGAAGCGAACCTTCT CGATGTA	52˚C	Primer Bank ID 4503979a1
βIII Tubulin	GCTCAGGGGCCTT TGGACATCTCTT	TTTTCACACTCCTTC CGCACCACATC	60˚C	(Mesquita et al., 2015)

Table S2. Nucleotide sequences of primers used in quantitative Real time PCR

Gene	Forward	Reverse	References
Vegf 165	ATCTTCAAGCCATCCTG TGTGC	CAAGGCCCACAGGGATTTTC	(Medford et al., 2009)
HIF1 α	CCAGCAGACTCAAATA CAAGAACC	TGTATGTGGGTAGGAGATGG AGAT	(Li et al., 2006)
NERF2	AGTGGATCTGCCAACT ACTC	CATCTACAAACGGGAATGTC TG	(Clements et al., 2006)
OXR1	CTGATGGTGATTAAAG ACAGTG	CACTTAAAGACCTCAAACTC C	Sigma (Gene ID-5504Catalog.no,KSPQ20 12G)
C3	TCACCGTCAACCACAA GCTGCTACC	TTTCATAGTAGGCTCGGATCT TCCA	(Wang et al., 2008)
IL1 β	AGCTGATGGCCCTAAA CAGA	GGAGATTCGTAGCTGGATGC	(Rushworth et al., 2011)
Caspase -3	ACATGGCGTGTCATAA AATACC	CACAAAGCGACTGGATGAAC	(Lan et al., 2014)
BAX	TGCTTCAGGGTTTCATC CAG	GGCGGCAATCATCCTCTG	(Savli et al., 2003)
CXCR4	AGCATGACGGACAAGT ACAGG	GATGAAGTCGGGAATAGTCA GC	(Vaday et al., 2004)
IL8	GACCACACTGCGCCAA CAC	CTTCTCCACAACCCTCTGCAC	(Filewod et al., 2009)
β Actin	TCTACAATGAGCTGCG TGTG	GGTGAGGATCTTCATGAGGT	(Horvatinovich et al., 1994)

Supplementary videos

Movie S1-S2: Measurement of intracellular Ca^{2+} transient in MRC using EVOS microscope (magnification 20X). Movie files show the Ca^{2+} spiking corresponding to no stress level (Movie S1) and Hypoxia (Movie S2) Spiking response was measured for 600 sec.

Supplementary Figures

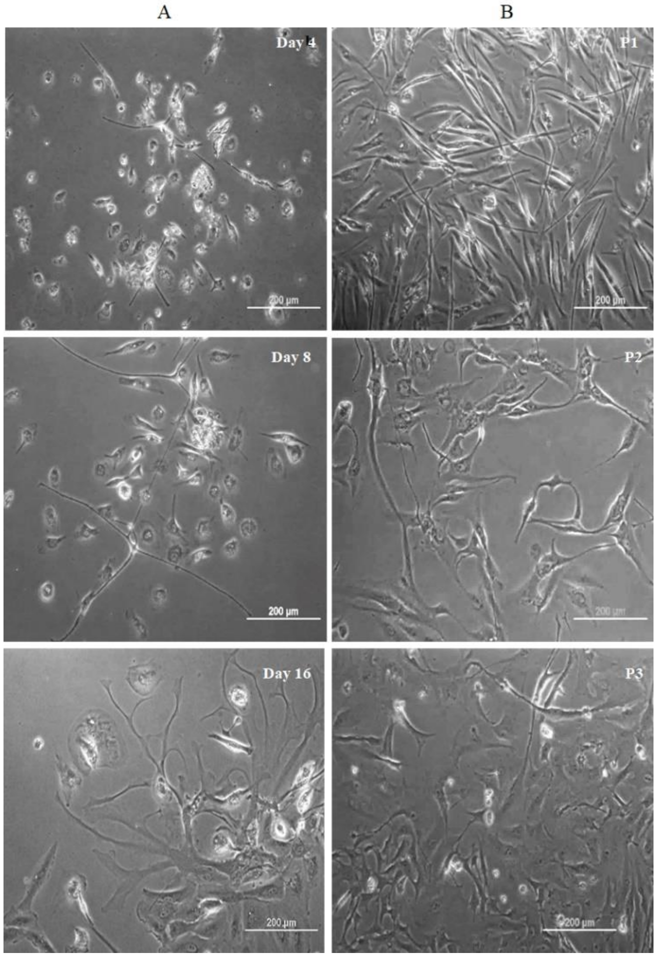

Supplementary Figure 1. Representative phase contrast images of cells cultured from retina of human cadaveric/ enucleated eyes. (**A**) Morphology of the cells after 4th, 8th and 16th days of culture at P0 stage. The cells showing the characteristic morphology of glial and neuronal type cells at day 16 confirm the heterogeneous retinal cell types in culture. (**B**) Morphology of cells in the MRC at 1st, 2nd and 3rd passages confirming the culture system is able to maintain these heterogeneous populations until 3rd passage (Magnification 10X, Scale bar 200 µm).

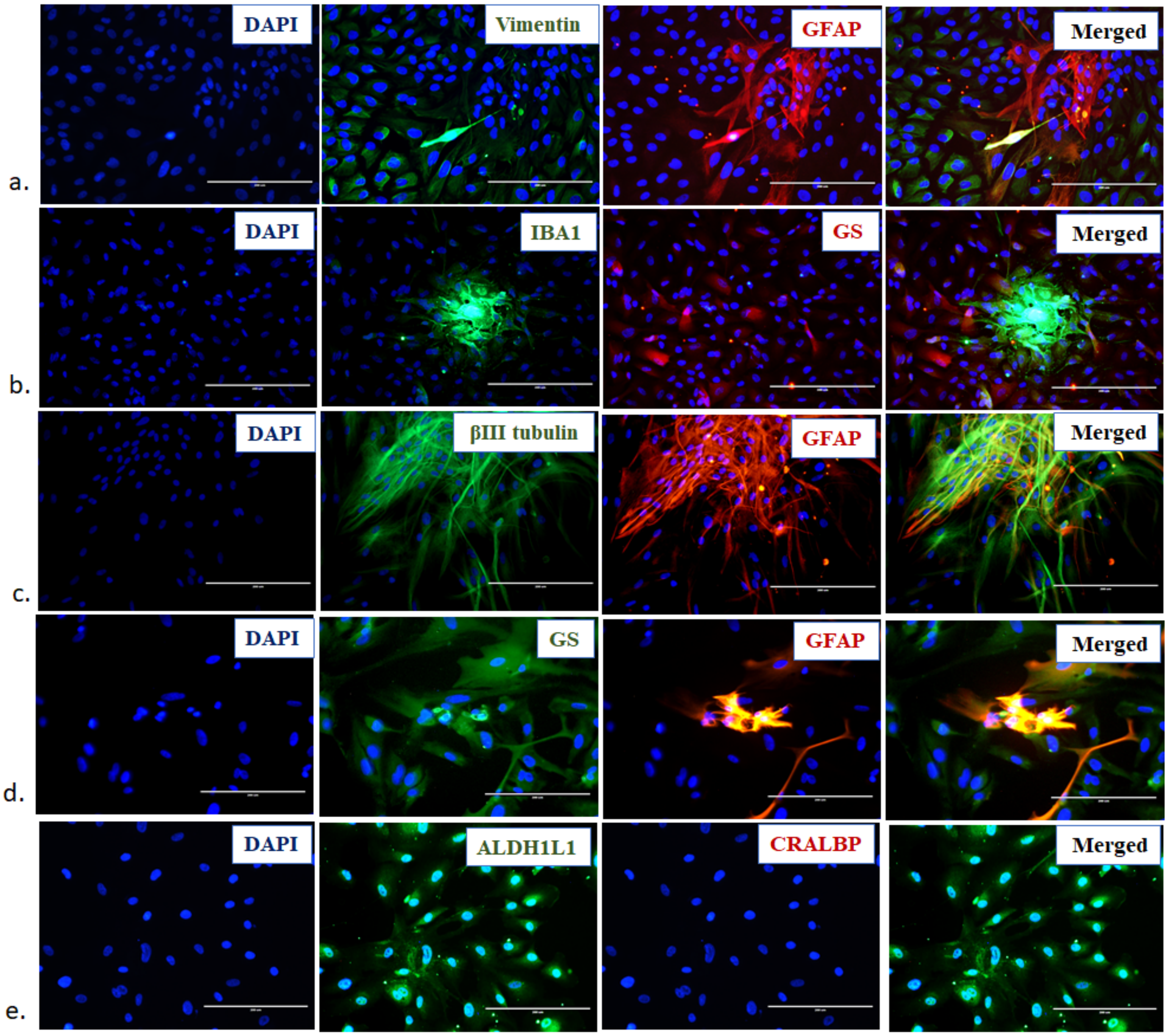

Supplementary Figure 2. Representative immunofluorescent images of cells in Mixed retinal culture. The images clearly showing interaction of different cell types in the developed culture system. Panel showing the co-staining of (a) vimentin (Müller glia) and GFAP (Astrocytes), (b) IBA-1 (Microglia) and GS (Müller glia), (c) β-III tubulin (Neurons) and GFAP (Astrocytes), (d) GS (Müller glia) and GFAP (Astrocytes) (e) ALDH1L1 (Astrocytes) and CRALBP (Muller glia, not worked) (Magnification- 20X, scale bar -200 µm). (Magnification 20X, scale bar, 200 µm).

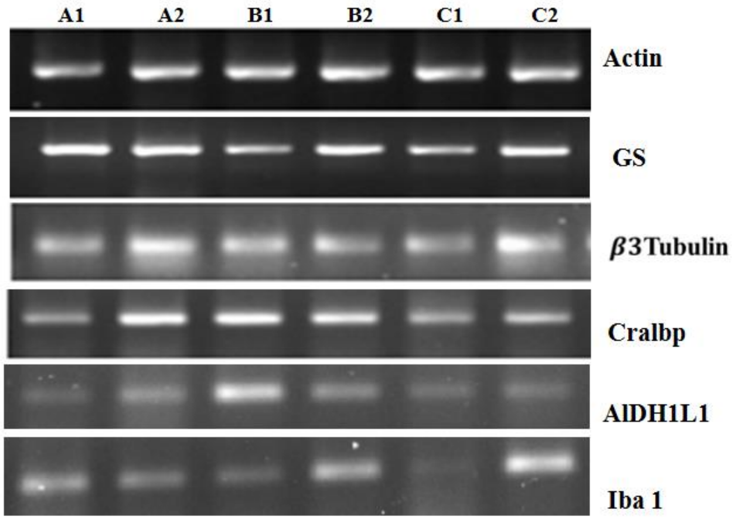

Supplementary Figure 3. Gene expression of neuron and glial cell specific markers from three different retinal donors' tissue at P1 and P2 passages, (A, B and C represents cells cultured from three different retinal tissues and 1 & 2 represents P1 and P2 passages)

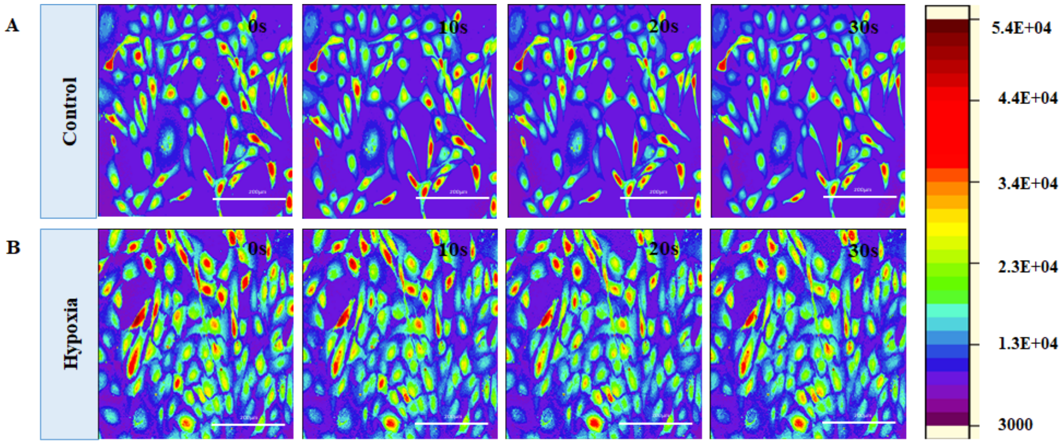

Supplementary Figure 4. Time lapse images of the spatial intensity mappings of cytosolic calcium transients in human primary mixed retinal culture (**A**) no stress (**B**) hypoxia (Magnification 20X, Scale bar 200 µm)

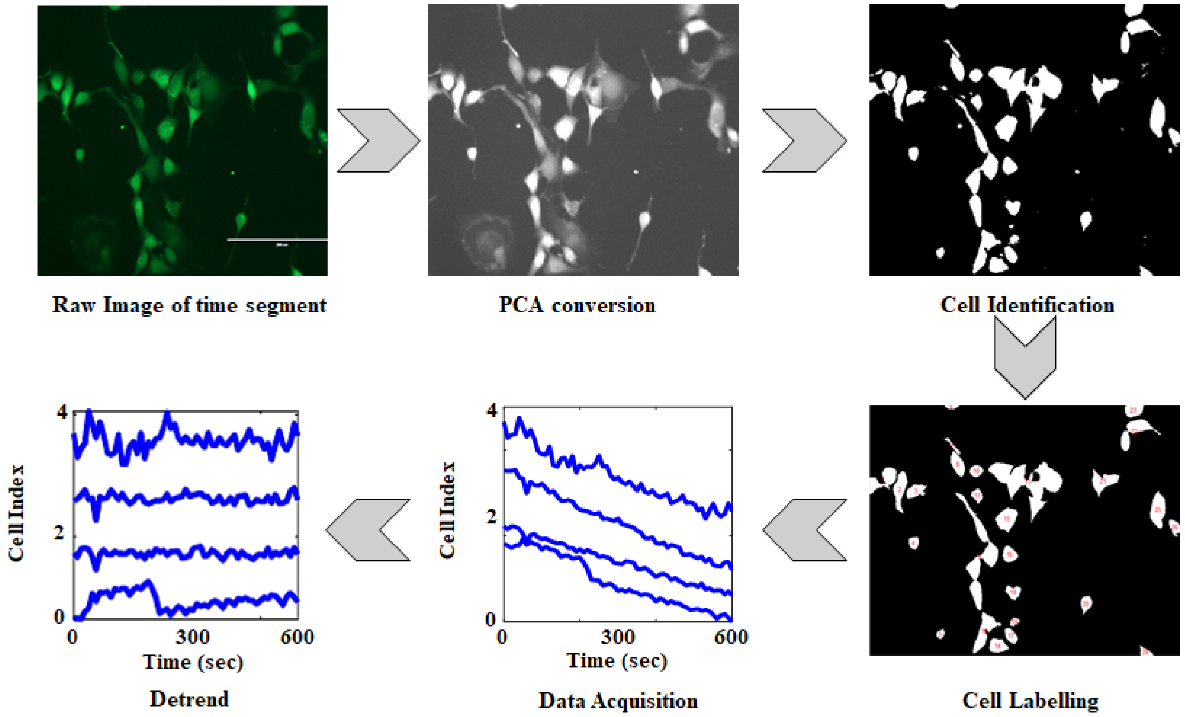

Supplementary Figure 5. Workflow representing various steps consisting of data acquisition, automated cell segmentation, cell labeling and data processing from the raw time-lapse videos

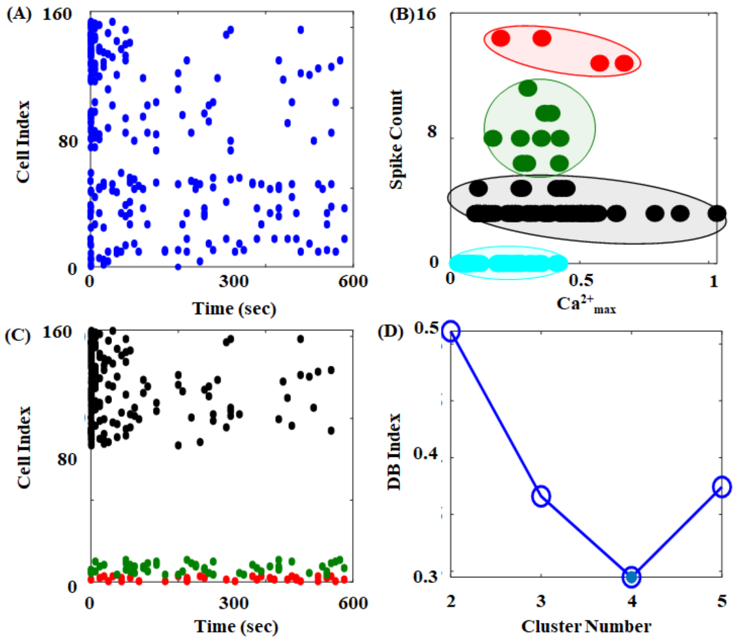

Supplementary Figure 6. k-means clustering of Ca^{2+} spiking in control MRC (**A**) Raster plots representing the network activity in MRC (**B**) Clustering of Ca^{2+} spiking train in a MRC population using two features, Ca^{2+} spike-count and maximum Ca^{2+} spiking amplitude (Ca^{2+}_{max}) (**C**) Raster plot showing the clustering pattern in MRC population (**D**) Identification of optimal number of clusters for the Ca^{2+} spiking train using Davies-Bouldin index

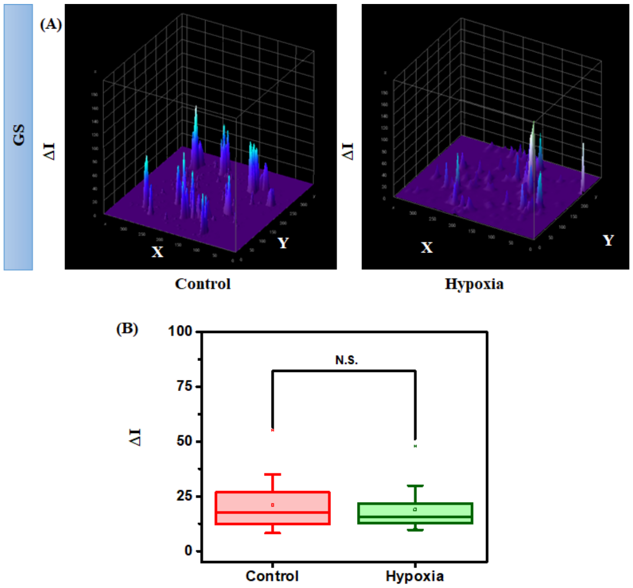

Supplementary Figure 7. (**A**) GS expression in MRC under no stress and hypoxia (**B**) Surface plot showing GS expression under no stress and hypoxia (**C**) Comparison of GS expression between no stress and hypoxia. N.S.: not significant.

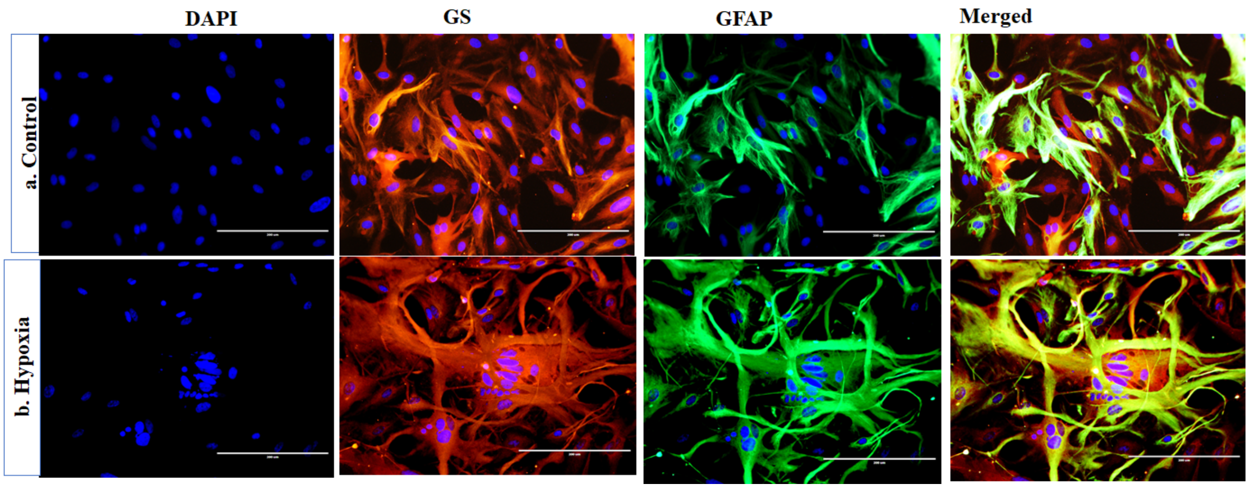

Supplementary Figure 8. Representative immunofluorescent images of GS and GFAP in cells under (a) control and (b) hypoxic conditions. (Magnification, 20X, Scale bar- 200 µm).

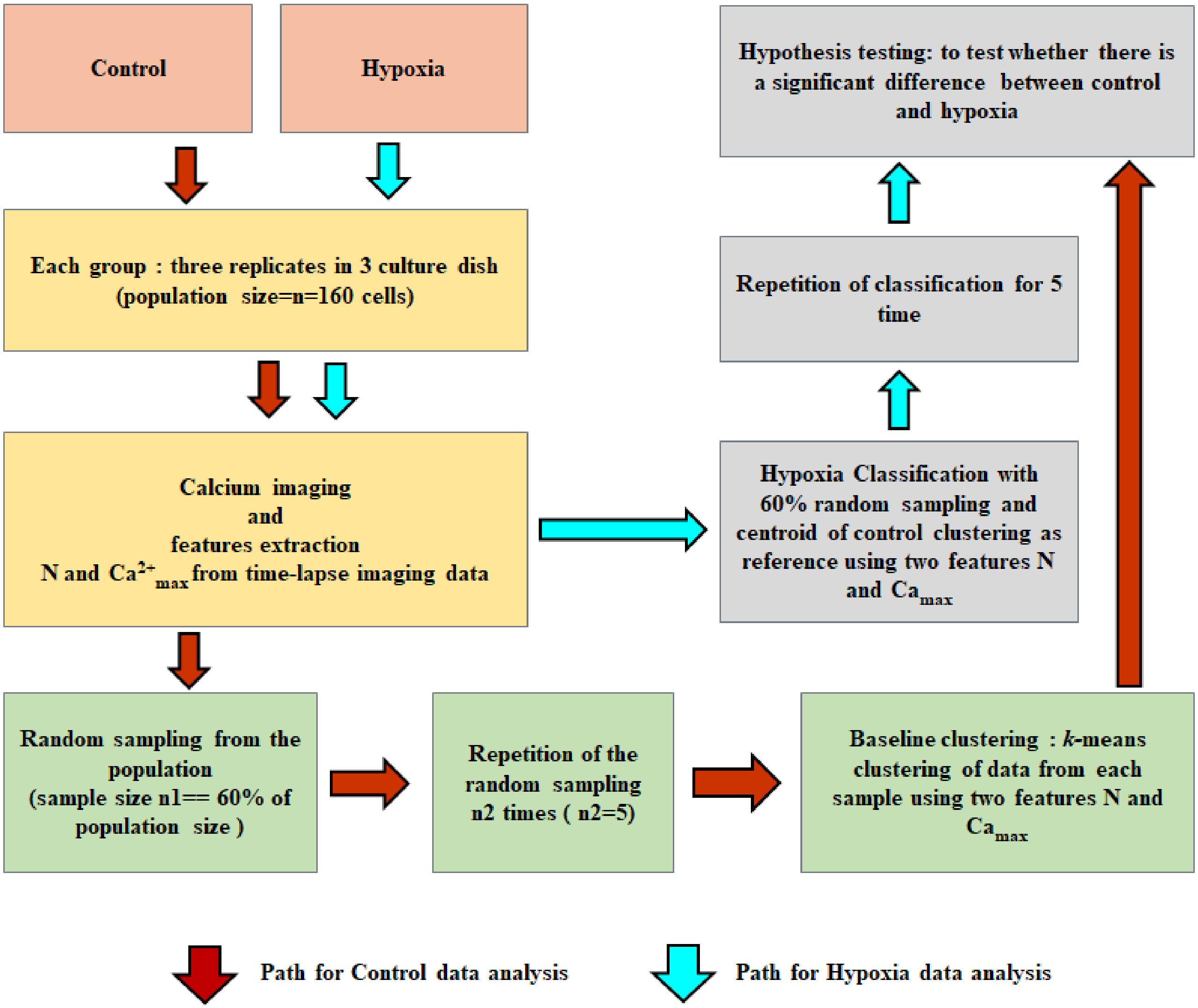

Supplementary Figure 9. A flow chart describing the detatiled summary of the Ca^{2+} imaging data analysis.

References

Clements, C.M., McNally, R.S., Conti, B.J., Mak, T.W., and Ting, J.P. (2006). DJ-1, a cancer- and Parkinson's disease-associated protein, stabilizes the antioxidant transcriptional master regulator Nrf2. *Proc Natl Acad Sci U S A* 103(41), 15091-15096. doi: 10.1073/pnas.0607260103.

Filewod, N.C., Pistolic, J., and Hancock, R.E. (2009). Low concentrations of LL-37 alter IL-8 production by keratinocytes and bronchial epithelial cells in response to proinflammatory stimuli. *FEMS Immunol Med Microbiol* 56(3), 233-240. doi: 10.1111/j.1574-695X.2009.00571.x.

Horvatinovich, J.M., Sparks, S.D., and Borowitz, M.J. (1994). Detection of terminal deoxynucleotidyl transferase by flow cytometry: a three color method. *Cytometry* 18(4), 228-230. doi: 10.1002/cyto.990180407.

Lan, W., Wan, S., Gu, W., Wang, H., and Zhou, S. (2014). Mechanisms behind the inhibition of lung adenocarcinoma cell by shikonin. *Cell Biochem Biophys* 70(2)**,** 1459-1467. doi: 10.1007/s12013-014-0083-5.

Lawrence, J.M., Singhal, S., Bhatia, B., Keegan, D.J., Reh, T.A., Luthert, P.J., et al. (2007). MIO-M1 cells and similar muller glial cell lines derived from adult human retina exhibit neural stem cell characteristics. *Stem Cells* 25(8)**,** 2033-2043. doi: 10.1634/stemcells.2006-0724.

Li, Q.F., Wang, X.R., Yang, Y.W., and Lin, H. (2006). Hypoxia upregulates hypoxia inducible factor (HIF)-3alpha expression in lung epithelial cells: characterization and comparison with HIF-1alpha. *Cell Res* 16(6)**,** 548-558. doi: 10.1038/sj.cr.7310072.

Medford, A.R., Douglas, S.K., Godinho, S.I., Uppington, K.M., Armstrong, L., Gillespie, K.M., et al. (2009). Vascular Endothelial Growth Factor (VEGF) isoform expression and activity in human and murine lung injury. *Respir Res* 10**,** 27. doi: 10.1186/1465-9921-10-27.

Mesquita, F.C., Kasai-Brunswick, T.H., Gubert Fde, M., Borgonovo, T., Silva-dos-Santos, D., de Araujo, D.S., et al. (2015). Generation of human iPS cell line ihFib3.2 from dermal fibroblasts. *Stem Cell Res* 15(3)**,** 445-448. doi: 10.1016/j.scr.2015.09.001.

Orsmark, C., Skoog, T., Jeskanen, L., Kere, J., and Saarialho-Kere, U. (2007). Expression of allograft inflammatory factor-1 in inflammatory skin disorders. *Acta Derm Venereol* 87(3)**,** 223-227. doi: 10.2340/00015555-0225.

Rushworth, S.A., Shah, S., and MacEwan, D.J. (2011). TNF mediates the sustained activation of Nrf2 in human monocytes. *J Immunol* 187(2)**,** 702-707. doi: 10.4049/jimmunol.1004117.

Savli, H., Sirma, S., Nagy, B., Aktan, M., Dincol, G., and Ozbek, U. (2003). Real-time PCR analysis of the apoptosis related genes in ATRA treated APL t(15;17) patients. *Exp Mol Med* 35(5)**,** 454-459. doi: 10.1038/emm.2003.59.

Vaday, G.G., Hua, S.B., Peehl, D.M., Pauling, M.H., Lin, Y.H., Zhu, L., et al. (2004). CXCR4 and CXCL12 (SDF-1) in prostate cancer: inhibitory effects of human single chain Fv antibodies. *Clin Cancer Res* 10(16)**,** 5630-5639. doi: 10.1158/1078-0432.CCR-03-0633.

Wang, J., Ohno-Matsui, K., Yoshida, T., Kojima, A., Shimada, N., Nakahama, K., et al. (2008). Altered function of factor I caused by amyloid beta: implication for pathogenesis of age-related macular degeneration from Drusen. *J Immunol* 181(1)**,** 712-720.
